# Deconvolution and compensation of mass spectrometric overlap interferences with the miniRUEDI portable mass spectrometer

**DOI:** 10.1016/j.mex.2020.101038

**Published:** 2020-08-21

**Authors:** Matthias S. Brennwald, Yama Tomonaga, Rolf Kipfer

**Affiliations:** a Department of Water Resources and Drinking Water, Eawag, Swiss Federal Institute of Aquatic Science and Technology, Dübendorf, Switzerland; b Department of Earth Sciences, Swiss Federal Institute of Technology in Zurich, Institute of Geochemistry and Petrology, Switzerland

**Keywords:** Isobaric ions, Environmental gases, ruediPy

## Abstract

The miniRUEDI is a portable mass spectrometer system designed for on-site analysis of gases in the environment during field work and at remote locations. For many gas species (e.g., He, Ar, Kr, N_2_, O_2_, CO_2_) the ion-current peak-heights measured with the mass spectrometer can usually be calibrated in terms of the partial pressures by simple peak-height comparison relative to a gas standard with well known partial pressures. However, depending on the composition of the analysed gases, the ion currents measured at certain *m*/*z* ratios may result from overlapping signals of multiple species (for example CH_4_, O_2_ and N_2_ at m/z=15 and 16; or Ne, Ar and H_2_O at m/z=20).

Here, we present a method extension to the existing miniRUEDI peak-height comparison in order to resolve such overlap interferences:

• We developed and tested a data processing procedure for accurate deconvolution and compensation of such mass-spectrometric overlap interferences.

• The method was incorporated into the miniRUEDI open-source software (ruediPy).

• The method substantially improves the analytical accuracy in situations where mass-spectrometric interferences cannot be avoided.

## Graphical abstract

**Figure:**
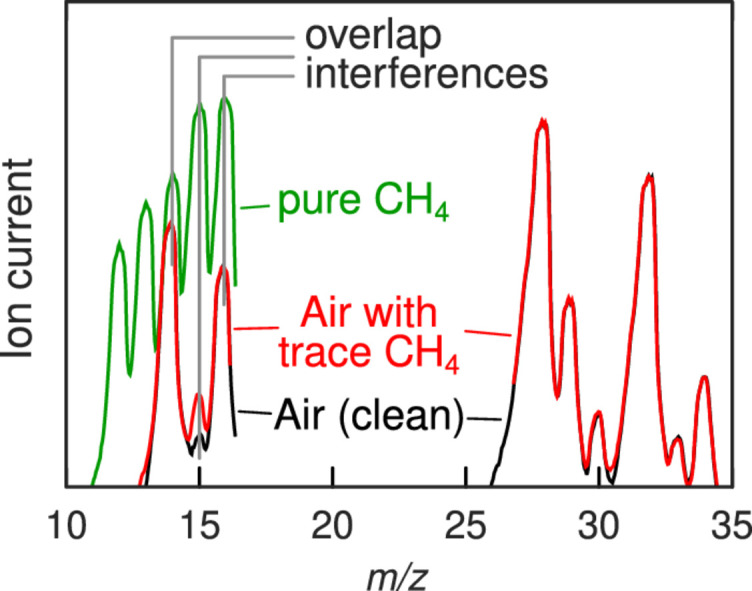

Specifications table

**Table:** 

Subject Area:	Environmental Science
More specific subject area:	On-site analysis of gases in the environment using a portable mass spectrometer
Method name:	miniRUEDI
Name and reference of original method:	M. S. Brennwald, M. Schmidt, J. Oser, R. Kipfer, A portable and autonomous mass spectrometric system for on-site environmental gas analysis, Environmental Science and Technology 50 (24) (2016) 13455–13463. doi:10.1021/acs.est.6b03669.
Resource availability:	https://github.com/brennmat/ruediPy

**Method details**

## Background

The miniRUEDI (Gasometrix GmbH, Switzerland) is a portable mass spectrometer system [1], [2], which is widely used in environmental research to study gas/water exchange processes, biogeochemical turnover, and the origin and transport of fluids. The miniRUEDI was designed as a simple and robust system for on-site gas analysis during field work at remote locations and allows quantification of individual gas species in gaseous or aqueous matrices. Since its development during the past decade [1], [2], the system has been used successfully to quantify the partial pressures of He, Ar, Kr, N_2_, O_2_ and CO_2_, in lakes, oceans, groundwaters, and a range of gaseous fluids in environmental systems [3], [4], [5], [6], [7], [8], [9], [10], [11], [12], [13], [14].

For quantification of the gas species, the miniRUEDI system uses a quadrupole mass spectrometer (Stanford Research Systems RGA200, *m*/*z* resolution ≤ 0.5 amu), which is equipped with a Faraday cup (FC) detector and an electron multiplier (EM) detector. The partial pressures of the different gas species are determined from the ion-current peak heights measured with the mass spectrometer by peak-height comparison relative to a reference gas with well known partial pressures of the species of interest [1]. This simple peak-height comparison relies on the assumption that each ion-current peak results from one single species only (the “target species” corresponding to that peak).

However, depending on the target species and the composition of the analysed gas mixture, some of the ion-current peaks involved in the analysis may result from different species contributing to the ion-current at the same *m*/*z* ratio (“overlap interference”). Such mass-spectrometric interferences cannot be used for peak-height comparison in a straight-forward way with the miniRUEDI. Unfortunately, avoiding the interferences is often not possible or impractical in many applications. For example, ^15^N${}^{+},$ ${}^{16}O^{+},$ and ${}^{16}O_{2}^{++}$ ions commonly interfere with the trace-level analysis of CH_4_ at m/z=15 or 16, and thereby hamper the analysis of this important greenhouse gas. Also, ${{}^{20}\left(H_{2}O\right)}^{+},$ ${}^{40}{\text{Ar}}^{++}$ and ${{\mathrm{CO}}_{2}}^{++}$ ions interfere with the analysis of atmospheric Ne, which is a sensitive natural proxy to study the recharge dynamics and aeration (“excess air” formation) of groundwaters [15]. Table 1 lists further details and other examples of mass-spectrometric interferences that are commonly encountered in miniRUEDI analyses of gases in environmental systems.

**Table 1:** Examples of mass-spectrometric overlap interferences commonly observed in environmental gas analysis.

Target species	*m*/*z*	Interferences	Notes
Ne	20	${}^{40}{\text{Ar}}^{++}$	Contribution of ${}^{40}{\text{Ar}}^{++}$ can be reduced by lowering ionisation energy, but this also lowers the sensitivity of the mass spectrometer and may therefore affect overall data quality.
	20	${{}^{20}\left(H_{2}O\right)}^{+}$	H_2_O is highly persistent in the mass-spectrometer vacuum.
	22	${{\mathrm{CO}}_{2}}^{++}$	^22^Ne analysis is often impossible because the m/z=22 ion current is dominated by ${{\mathrm{CO}}_{2}}^{++}$.
CH_4_	16	${}^{16}O^{+},$${}^{16}{O_{2}}^{++}$	CH_4_ analyis is often impossible on m/z=16 due to the large ${}^{16}O^{+}$ and ${}^{16}{O_{2}}^{++}$ ion currents.
	15	${}^{15}N^{+}$	Analyse CH_4_ via its ${\text{CH}}_{3}^{+}$ fragment on m/z=15. Potential interferences: fragments of N_2_ molecules containig ${}^{15}N^{+}$ and peak-tails from m/z=14 and m/z=16.
N_2_	14, 28	${\text{CO}}^{++},$${\text{CO}}^{+}$	${\text{CO}}^{+}$ fragments from CO_2_ are usually not relevant in air-like gases.
H_2_S	34	${{}^{34}\left(O_{2}\right)}^{+}$	H_2_S abundance is typically very low, so even small amounts of residual O_2_ may affect environmental H_2_S analysis.
C_3_H_8_	44	${{\mathrm{CO}}_{2}}^{+}$	m/z=44 ion current is usually dominated by ${{\mathrm{CO}}_{2}}^{+}$.
	43	(CO_2_)	Analyse C_3_H_8_ via its C_3_H_7_ fragment on m/z=43. With CO_2_ rich gases, the peak-tail from m/z=44 may interfere.
N_2_O	44	${{\mathrm{CO}}_{2}}^{+}$	m/z=44 ion current is usually dominated by ${{\mathrm{CO}}_{2}}^{+}$.
	30	various	Analyse N_2_O via its NO fragment on m/z=30; interferences with C^18^O${}^{+},$ hydrocarbon fragments, and potentially also the tails of the N_2_ (m/z=28) and O_2_ (m/z=32) peaks.

If such interferences cannot be avoided, they need to be disentangled and quantified before peak-height comparison for calibration of the partial pressures. To this end, the relative fractions of the interfering ion currents are deconvolved in terms of the species involved in the interference [16]. The deconvolution yields the ion-current fractions pertaining to the target species, and therefore allows accurate peak-height comparison even in the presence of mass-spectrometric interferences.

Here, we present an extension to the existing miniRUEDI peak-height comparison technique. This method extension deconvolves and quantifies mass-spectrometric interferences in miniRUEDI analyses, and thereby substantially improves the analytical accuracy in situations where mass-spectrometric interferences cannot be avoided. To facilitate the adoption of the method in field applications of the miniRUEDI, the procedures for mass-spectrometric deconvolution and interference compensation were integrated in the ruediPy open-source software toolbox for instrument control and data processing with the miniRUEDI [17].

## Deconvolution of mass-spectrometric interferences

Consider the mass spectrum of ion-current peak-heights *y*(*m*/*z*) observed with an arbitrary gas mixture consisting of *N* different gas species. This mass spectrum is modelled as a linear combination $\tilde{y}\left(m/z\right)$ of basis spectra *x_i_*(*m*/*z*) (i=1,…,N) [16]. The requirements for the *x_i_* are that they are linearly independent and well known from external analysis. While the *x_i_* typically correspond to pure gases containing only one single species, it may also be practical to consider gas mixtures containing different species (see Section 4 for examples). For convenience, the *x_i_* are defined as dimensionless spectra that are normalised such that $\max_{m/z}\left\{x_{i}\left(m/z\right)\right\}=1$.

The peak heights $\tilde{y}\left(m/z\right)$ are given by the following equation system ($m/z=\mu_{1},\ldots ,\mu_{M}$):

(1) $$\begin{array}{ccccccccc} \tilde{y}\left(\mu_{1}\right) & = & a_{1}x_{1}\left(\mu_{1}\right) & + & a_{2}x_{2}\left(\mu_{1}\right) & + & \cdots & + & a_{N}x_{N}\left(\mu_{1}\right)\\ \tilde{y}\left(\mu_{2}\right) & = & a_{1}x_{1}\left(\mu_{2}\right) & + & a_{2}x_{2}\left(\mu_{2}\right) & + & \cdots & + & a_{N}x_{N}\left(\mu_{2}\right)\\ \vdots & & & & & & & & \vdots \\ \tilde{y}\left(\mu_{M}\right) & = & a_{1}x_{1}\left(\mu_{M}\right) & + & a_{2}x_{2}\left(\mu_{M}\right) & + & \cdots & + & a_{N}x_{N}\left(\mu_{M}\right) \end{array}$$

If *N* ≤ *M*, the relative contributions ($a_{1},\ldots ,a_{N}$) of the basis spectra to the spectrum observed with an arbitrary gas mixture can be estimated from the above equation system (“spectral deconvolution”) [16]. To this end, the best-fit solution of the equation system is determined by minimising the sum *χ*^2^ of the squared error-weighted residuals of the model relative to the observed data (error-weighted least-squares regression) [18]:

$$\chi^{2}=\sum_{j=1}^{M}{\left(\frac{\tilde{y}\left(\mu_{j};a_{1},\ldots ,a_{N}\right)-y\left(\mu_{j}\right)}{\Delta y(\mu_{j})}\right)}^{2}$$

Note that the *y*(*μ_j_*) are determined as the means of repeated peak-height measurements. The Δ*y*(*μ_j_*) are therefore, in a first step, estimated from the error of the mean [1]. Note that the error of the mean tends to underestimate the true error because it only captures the random noise during a single measurement, but does not account for additional uncertainties such as those related to instrumental drift or non-linearities of the mass spectrometer. The Δ*y*(*μ_j_*) are therefore heuristically enforced to a minimum value of 1 %, which corresponds to the typical peak-height error that can be achieved with the miniRUEDI [1].

The standard errors Δ*a_i_* of the *a_i_* are, in a first step, estimated by error propagation of the Δ*y*(*μ_j_*) in the *χ*^2^ regression. In a second step, the *χ*^2^ value is used to quantify the overall difference between the modeled and the observed peak heights (i.e., the goodness of the regression fit). If $\chi^{2}≲M-N,$ the difference between the modeled and the observed spectra is fully explained by the Δ*y*(*μ_j_*). If $\chi^{2}>>M-N,$ either the model is unsuitable to explain the observed peak heights (i.e., the equation system (1) is incomplete), or the Δ*y*(*μ_j_*) were underestimated by a factor $\sqrt{\chi^{2}/\chi_{\sigma }^{2}},$ where $\chi_{\sigma }^{2}$ is the *χ*^2^ value corresponding to the 1-*σ* quantile. In the latter case, the Δ*a_i_* are therefore rescaled in a second step to account for the full uncertainty of the data:

$$\chi^{2}>\chi_{\sigma }^{2}\;\Rightarrow \;\text{replace}\;\text{all}\;\Delta a_{i}\;\text{by}\;\sqrt{\frac{\chi^{2}}{\chi_{\sigma }^{2}}}\times \Delta a_{i}$$

## Compensation of mass-spectrometric interferences

For analysis of a given target species at a given $m/z=\mu_{k},$ the corresponding interferences need to be quantified and subtracted from the measured ion current at this *m*/*z* ratio. The fraction of the ion-current contributed by the target species to the total ion current at *μ_k_* is computed from *k*th line of equation system (1) using the *a_i_* and the Δ*a_i_* values as determined by the deconvolution procedure (Section 2). Only the ion-current fraction contributed by the target species is used in the peak-height comparison to quantify the partial pressure of the target species.

## Demonstration and validation examples

The performance of the deconvolution method for interference compensation is demonstrated using two examples related to typical applications in environmental research.

### Example-1: CH_4_ analysis on m/z=15

The analysis of CH_4_ in environmental gases is typically affected by mass-spectrometric interferences on m/z=15 and m/z=16 (see Table 1). The m/z=16 ion current is commonly dominated by O${}^{+}$ and $O_{2}^{++}$ and is therefore hardly useful for CH_4_ analysis. As a way out, CH_4_ is analysed via its ${\mathrm{CH}}_{3}^{+}$ fragment at m/z=15. The ion current at this *m*/*z* ratio is affected to a considerably lesser degree by the interferences of ${{}^{\text{15}}\text{N}}^{+}$ and peak-tails from m/z=14 and m/z=16.

To illustrate the analysis of CH_4_ and interference compensation at m/z=15, two test-gas mixtures are considered in this example. The nominal gas compositions of these gases are:

• Gas-I: 23.1% (vol) CH_4_ in N_2_ (Messer AG, Switzerland)
• Gas-II: 250 ± (13)ppm (vol) CH_4_ in air (Isometric Instruments, Canada)

**Fig. 1:**
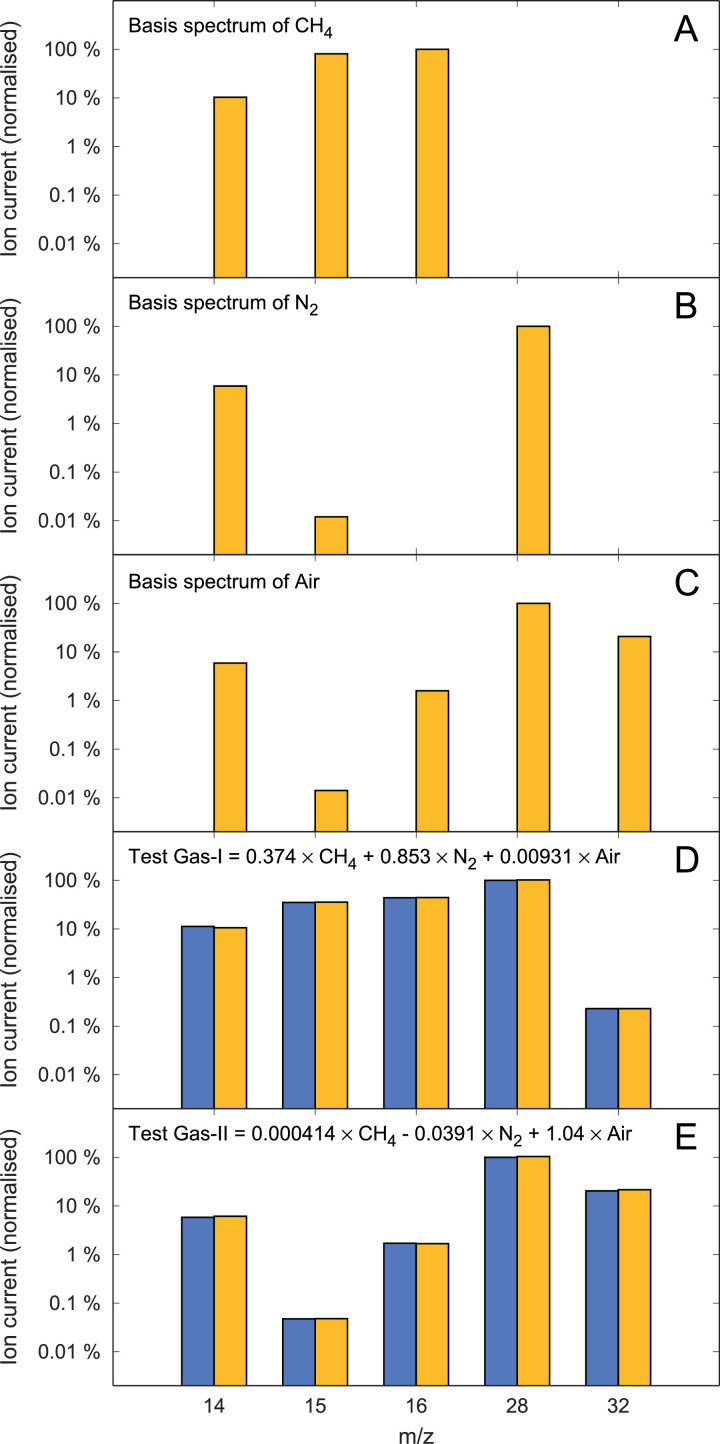
Normalised ion-current spectra and deconvolution of Example-1. The yellow bars in panels A–C show the basis spectra used in the deconvolution (CH_4_, N_2_ and Air; see Table 3). Panels D and E show the spectra of Gas-I and Gas-II as determined in the measurements (blue bars) and by deconvolution in terms of the basis spectra (yellow bars; see Table 2). Note the logarithmic scaling of the ion-current data.

The ion currents measured with these test gases using the detector at m/z=14,15,16,28,32 are listed in Table 2. These ion-current spectra were deconvolved in terms of the basis spectra of CH_4_, N_2_ and clean air (Table 3). The deconvolution is illustrated in Fig. 1. Note the remarkably good agreement of the deconvolution model results with the measured spectra of Gas-I and Gas-II.

**Table 2:** Deconvolution Example-1 (CH_4_ analysis): ion-current peak heights measured with test gases I and II using the Faraday cup, and relative fractions of the CH_4_ contribution to the ion currents as determined by deconvolution (see text for details).

*m*/*z*	Measured ion current (pA)		Relative CH_4_ fraction	
	Gas-I	Gas-II	Gas-I	Gas-II
14	127 ± 2	77.6 ± 0.9	(43 ± 1) %	(0.073 ± 0.003) %
15	394 ± 6	0.629 ± 0.008	(100 ± 2) %	(70 ± 3) %
16	491 ± 5	22.7 ± 0.1	(100 ± 2) %	(2.47 ± 0.09) %
28	1123 ± 8	1327 ± 6	–	–
32	2.6 ± 0.9	271 ± 5	–	–

**Table 3:** Basis spectra examples for CH_4_, N_2_, Ar, CO_2_ and clean air measured with a miniRUEDI (normalised ion currents, measured with 70 eV ion-source electron-energy). Empty values are treated as zero.

*m*/*z*	Ion current (normalised)		
	CH_4_	N_2_	Air
14	0.103	0.059	0.059
15	0.806	0.00012	0.00014
16	1.0		0.0158
28		1.0	1.0
32			0.208

For Gas-I, the relative contribution of CH_4_ to the ion current at m/z=15 as determined by the deconvolution method is 100 ± (2)% (Table 2). It is not surprising that CH_4_ dominates the m/z=15 ion current, because the CH_4_ concentration in Gas-I is rather high.

For Gas-II, however, the relative contribution of CH_4_ to the m/z=15 ion current is only 70 ± (3)% (Table 2). The remaining 30 ± (2)% are attributed to the air basis spectrum, which must be subtracted from the m/z=15 ion current for CH_4_ quantification.

To validate these deconvolution results, Gas-II is treated as an unknown sample and Gas-I is used as a reference standard to calibrate the CH_4_ analysis. The ratio of the CH_4_ concentrations in Gas-I and Gas-II is equal to the ratio of the respective ion currents. Therefore, with ${\left[{\mathrm{CH}}_{4}\right]}_{I}=23.1$ % and using the data in Table 2, the CH_4_ concentration in Gas-II is determined as follows:

Raw ion-current peak heights (without interference compensation):

$${\left[{\mathrm{CH}}_{4}\right]}_{\text{II},\text{raw}}\;=\;\frac{(0.629\pm 0.008)\mathrm{pA}}{(394\pm 6)\mathrm{pA}}\;\times \;23.1\%\;=\;\left(369\pm 7\right)\;\mathrm{ppm}$$

CH_4_ ion-current peak heights (with interference compensation):

$${\left[{\mathrm{CH}}_{4}\right]}_{\text{II},\text{comp}}\;=\;\frac{(0.70\pm 0.03)\times (0.629\pm 0.008)\mathrm{pA}}{(1.00\pm 0.02)\times (394\pm 6)\mathrm{pA}}\times 23.1\%\;=\;\left(260\pm 13\right)\;\mathrm{ppm}$$

The CH_4_ concentration value in Gas-II [CH_4_]_II,raw_ as calculated from the raw m/z=15 ion currents is not consistent with the nominal CH_4_ concentration of (250 ± 13) ppm in Gas-II, because the interferences at m/z=15 were ignored in the calculation. However, if the interference compensation is applied to the m/z=15 ion currents, the resulting CH_4_ concentration value [CH_4_]_II,comp_ is in good agreement with the nominal CH_4_ concentration of Gas-II. Note that [CH_4_]_II,comp_ exhibits a slightly larger standard error than [CH_4_]_II,raw_. The increase in the error reflects the additional uncertainties introduced with the deconvolution, which must always be taken into account to assess the quality of the interference compensation.

### Example-2: Ne analysis on m/z=20

The analysis of Ne in environmental gases is typically affected by mass-spectrometric interferences on m/z=20 and m/z=22 (see Table 1). The m/z=22 ion current is commonly dominated by the omnipresent ${\mathrm{CO}}_{2}^{++}$ and is therefore not useful for ^22^Ne analysis. Ne is therefore quantified via the ^20^Ne isotope on m/z=20. However, the ion current at this *m*/*z* ratio is commonly subject to two interferences by ^40^Ar${}^{++}$ and ${{}^{20}\left(H_{2}O\right)}^{+}$. The ${}^{40}{\text{Ar}}^{++}$ interference was alleviated by setting the ionisation energy to 45 eV to prevent double ionisation of ^40^Ar [1]. The ${{}^{20}\left(H_{2}O\right)}^{+}$ interference cannot be avoided, because H_2_O is highly persistent in the mass-spectrometer vacuum and drying the gas before inlet to the mass spectrometer therefore does not help.

To illustrate Ne analysis and interference compensation on m/z=20, three test-gas mixtures are considered in this example:

• Gas-III: Dry synthetic air (N_2_ and O_2_ at 8:2 mixing ratio) with noble-gas spikes (Air Liquide, Switzerland):

326 ppm (vol) ^20^Ne

314 ppm (vol) ^36^Ar

9.27 % (vol) ^40^Ar

• Gas-IV: Same as Gas-III, but humid

• Gas-V: Atmospheric air with Ar spike:

15.8 ppm (vol) ^20^Ne

166 ppm (vol) ^36^Ar

4.91 % (vol) ^40^ Ar

The ion currents measured with these test gases using the EM detector at m/z=17,20,36 are listed in Table 4. These ion currents were deconvolved in terms of the basis spectra of H_2_O, Ne, and Ar using the basis data given in Table 5.

**Table 4:** Deconvolution Example-2 (Ne analysis): ion-current peak heights measured with test gases III, IV and V using the electron multiplier detector, and relative fractions of the ^20^Ne contribution to the ion currents as determined by deconvolution (see text for details).

*m*/*z*	Measured ion current (nA)			Relative Ne fraction		
	Gas-III	Gas-IV	Gas-V	Gas-III	Gas-IV	Gas-V
17	24.0 ± 0.1	96.4 ± 0.4	20.6 ± 0.1			
20	0.748 ± 0.008	1.37 ± 0.02	0.197 ± 0.002	(74 ± 1) %	(42 ± 1) %	(14 ± 2) %
36	4.59 ± 0.02	4.91 ± 0.02	2.29 ± 0.01

**Table 5:** Basis spectra examples for ^20^Ne, H_2_O, and Ar measured with a miniRUEDI (normalised ion currents, measured with 45 eV ion-source electron-energy). Empty values are treated as zero.

*m*/*z*	H_2_O	^20^Ne	Ar
17	0.164		
18	1.0		
20	1.34 × 10^−3^	1.0	1.06 × 10^−9^
36			3.00 × 10^−3^
40			1.0

Note that Ne analysis at m/z=20 in air-like gases requires the use of the EM detector, because the FC sensitivity is too low. In contrast, the high ion currents at m/z=18 (H_2_O main peak) and m/z=40 (Ar main peak) would saturate the EM detector. However, the FC ion currents at m/z=18 and 40 were not used in the deconvolution, because mixed EM and FC data were found to be unsuitable for deconvolution due to the drift in the EM/FC sensitivity ratio between different analysis steps.

Table 4 shows the deconvolution results for Gas-III, IV and V. For Gas-III, Ne contributes (74 ± 1) % to the ion current at m/z=20. For Gas-IV with its increased H_2_O partial pressure, the m/z=20 ion current is almost twice as high as in Gas-III, and the relative Ne contribution amounts to (42 ± 1) % only. For Gas-V, which exhibits a considerably lower Ne concentration, the Ne contribution to the m/z=20 ion current amounts to a mere (14 ± 2) %.

To validate these deconvolution results, Gas-IV and Gas-V are treated as unknown samples, and Gas-III is used as a reference to calibrate the Ne analysis (analogous to Example-1):

Raw ion-current peak heights (without interference compensation):

$${\left[{}^{20}\mathrm{Ne}\right]}_{\text{IV},\text{raw}}\;=\;\frac{(1.37\pm 0.02)\;\mathrm{nA}}{(0.748\pm 0.008)\;\text{nA}}\;\times \;326\;\mathrm{ppm}\;=\;\left(596\pm 10\right)\;\mathrm{ppm}$$

$${\left[{}^{20}\mathrm{Ne}\right]}_{V,\text{raw}}\;=\;\frac{(0.197\pm 0.002)\;\mathrm{nA}}{(0.748\pm 0.008)\;\mathrm{nA}}\;\times \;326\;\mathrm{ppm}\;=\;\left(86\pm 1\right)\text{ppm}$$

^20^Ne ion-current peak heights (with interference compensation):

$${\left[{}^{20}\mathrm{Ne}\right]}_{\text{IV},\text{comp}}\;=\;\frac{(0.42\pm 0.01)\times (1.37\pm 0.02)\;\text{nA}}{(0.74\pm 0.01)\times (0.748\pm 0.008)\;\text{nA}}\times 326\;\mathrm{ppm}\;=\;\left(338\pm 14\right)\;\text{ppm}$$

$${\left[{}^{20}\mathrm{Ne}\right]}_{V,\text{comp}}\;=\;\frac{(0.14\pm 0.02)\times (0.197\pm 0.002)\;\text{nA}}{(0.74\pm 0.01)\times (0.748\pm 0.008)\text{nA}}\times 326\;\text{ppm}\;=\;\left(16\pm 2\right)\;\text{ppm}$$

Similar to Example-1, the ^20^Ne concentration values calculated from the raw m/z=20 ion currents are far higher than the nominal ^20^Ne concentrations of the respective test gases. If the interference compensation is taken into account, the ^20^Ne concentration values are in good agreement with the nominal concentrations.

**Fig. 2:**
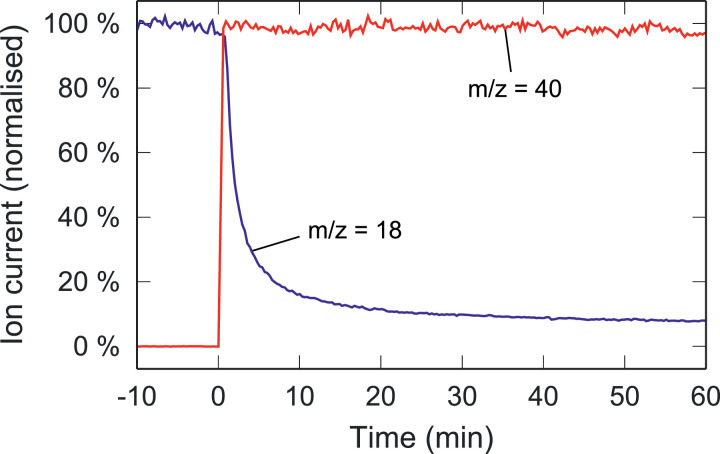
Ion currents at m/z=18 (blue) and m/z=40 (red) showing the transition when switching the gas inlet from humid N_2_ (*t* < 0 min) to dry air (*t* ≥ 0 min). The slow change of the m/z=18 ion current illustrates the persistence of H_2_O in the vacuum system.

Note that H_2_O tends to adsorb onto metal surfaces of the vacuum system and is therefore highly persistent in the miniRUEDI mass spectrometer. While the ion currents of other species is immediately controlled by their concentrations in the gases introduced into mass spectrometer, the H_2_O ion current therefore takes much longer to stabilise after switching the miniRUEDI gas inlet from one gas to another (Fig. 2). The H_2_O ion current must be stable during a given analysis step in order to allow robust compensation of the H_2_O interference. After switching between gas inlets, the H_2_O ion current was therefore allowed to stabilise for 20 min before starting a new measurement in the above example. Depending on the application, such long idle times may conflict with the requirements for the duty cycle and time resolution for the gas analysis.

## Implementation in the miniRUEDI software

The deconvolution and interference compensation of the raw ion-current data have been integrated in the ruediPy open-source software [17]. The procedures are implemented as a data pre-processing tool, which can be applied if mass-spectrometric interferences cannot be avoided in the conventional peak-height comparison method. After pre-processing, the interference-corrected ion currents are processed using the existing standard protocol for partial-pressure calibration by peak-height comparison [1].

### Gas analysis

The analysis procedure follows the existing standard procedures implemented for each miniRUEDI analysis step (sample, standard, or blank) in the ruediPy software, whereby the following extensions for the deconvolution tool were added:

• In addition to the normal ion-current measurements (PEAK and ZERO readings), optional “helper” peak-height measurements may be recorded at additional *m*/*z* ratios in order to provide further constraints the regression equation system (1) (Section 2). Such “helper” readings are marked by the software as PEAK_DECONV and ZERO_DECONV; they are not used in the peak-height comparison to calculate the partial pressures (Section 3).
• For every target *m*/*z* ratio that requires interference compensation by deconvolution, a data block with the corresponding deconvolution parameters is written to the data file (marked as DECONVOLUTION, see Section 5.2).

### The DECONVOLUTION block

Each DECONVOLUTION block contains the following data fields (see example below for the data format):

• target_mz (integer): the *m*/*z* ratio where the ion-current needs to be compensated for mass-spectrometric interferences.
• target_species (string): name/label of the target gas species for which the partial pressure will be quantified using the compensated ion current at target_mz.
• detector (character F or M): detector used for the ion-current measurement.
• MS_EE (integer or real number): electron-energy setting of the ion source during analysis. The MS_EE field is provided only for user documentation of the ion-source settings; it is not used in the data processing of the deconvolution tool.
• basis (array): basis spectra (normalised ion currents) to be used in the deconvolution.

The following DECONVOLUTION block is an example for CH_4_ analysis on m/z=15 with compensation of interferences resulting from N_2_ and O_2_ as described in Example-1 (Section 4.1):

DECONVOLUTION:

target_mz=15 ;

target_species=CH4 ;

detector=F ;

MS_EE=70 eV ;

basis=(

(’CH4’, 14,0.103, 15,0.806, 16,1.0),

(’N2’, 14,0.059, 15,0.00012, 28,1.0),

(’AIR’, 14,0.059, 15,0.00014, 16,0.0158, 28,1.0, 32,0.208))

#### Notes:

• In the ruediPy data files, the DECONVOLUTION block is written on a single data line.
• The basis spectra are specific to the mass-spectrometer instrument and the electronic settings of the ion source. It is therefore recommended to measure the basis spectra using the same mass spectrometer and ion-source settings as used with the gas analysis.
• It is recommended to configure the interference compensation in the software before running the analysis in order to make sure that the correct DECONVOLUTION block is written automatically to the data files. However, it is also possible to add or modify DECONVOLUTION blocks in existing raw data files using an ASCII editor.

### Data processing

The data processing procedure implemented in the ruediPy software is as follows (extensions to existing standard procedures are marked in *italics*):

For each analysis step (sample, standard, blank):

• Read the raw data file of the analysis step.
• Determine the main ion-current peak-heights from the PEAK and ZERO readings (mean or median values)
• *Determine the helper peak-heights from the PEAK_DECONV and ZERO_DECONV helper readings (if any).*
• *For each DECONVOLUTIONblock (if any):*
  - *Deconvolution (*Section 2*): deconvolve all ion-current peak-height data according to the DECONVOLUTION parameters and determine the relative contributions of the spectrometric interferences at m/z  = target_mz using the peak-height data recorded with the detector specified in the detector field.*
  - *Interference compensation (*Section 3*): subtract all interferences determined in the deconvolution step from the detector ion-current peak-height determined at target_mz.*

*Calibration of partial pressures:* Convert the *(interference-corrected)* ion-current peak heights as determined from the PEAK and ZERO data to partial pressures using the existing peak-height comparison procedure [1].

## Conclusions

We present a method to deconvolve and compensate mass-spectrometric overlap interferences in miniRUEDI gas analyses. The procedures for deconvolution and compensation of the interferences were integrated in the open source software for miniRUEDI instrument control and data processing [17] in order to facilitate the adoption of the new method in miniRUEDI field applications.

The method was shown to substantially improve the analytical accuracy in situations where mass-spectrometric interferences cannot be avoided, and thereby expands the scientific application area for the miniRUEDI. In a first example, we validated the interference compensation for accurate trace-level CH_4_ analysis in air-like gas matrices, which expands the application range of the miniRUEDI in studies on the sources and the dynamics of this important greenhouse gas in environmental systems. In a second example, we demonstrated the compensation of interferences involved in the trace-level analysis of Ne in air-like gas matrices, which expands the analytical potential to study the linkages between groundwater recharge dynamics and the quality of water resources.

Note that the deconvolution method for interference compensation with the miniRUEDI is not conceptually linked to specific types of target gas species or gas matrices. Therefore, as long as the species involved in a given interference are known and the corresponding basis spectra can be measured, the method is in principle applicable to any target gas species or gas matrix, and thereby substantially expands the application range of the miniRUEDI.

## Declaration of Competing Interest

The authors declare that they have no known competing financial interests or personal relationships that could have appeared to influence the work reported in this paper.
